# Deposition pattern and subcellular distribution of disease-associated prion protein in cerebellar organotypic slice cultures infected with scrapie

**DOI:** 10.3389/fnins.2015.00410

**Published:** 2015-11-04

**Authors:** Hanna Wolf, André Hossinger, Andrea Fehlinger, Sven Büttner, Valerie Sim, Debbie McKenzie, Ina M. Vorberg

**Affiliations:** ^1^German Center for Neurodegenerative DiseasesBonn, Germany; ^2^Centre for Prions and Protein Folding Diseases, University of AlbertaEdmonton, AB, Canada; ^3^Department of Neurology, Rheinische Friedrich-Wilhelms-University of BonnBonn, Germany

**Keywords:** prion, organotypic slice culture, scrapie, PrP, guanidine hydrochloride denaturation

## Abstract

Organotypic cerebellar slices represent a suitable model for characterizing and manipulating prion replication in complex cell environments. Organotypic slices recapitulate prion pathology and are amenable to drug testing in the absence of a blood-brain-barrier. So far, the cellular and subcellular distribution of disease-specific prion protein in organotypic slices is unclear. Here we report the simultaneous detection of disease-specific prion protein and central nervous system markers in wild-type mouse cerebellar slices infected with mouse-adapted prion strain 22L. The disease-specific prion protein distribution profile in slices closely resembles that *in vivo*, demonstrating granular spot like deposition predominately in the molecular and Purkinje cell layers. Double immunostaining identified abnormal prion protein in the neuropil and associated with neurons, astrocytes and microglia, but absence in Purkinje cells. The established protocol for the simultaneous immunohistochemical detection of disease-specific prion protein and cellular markers enables detailed analysis of prion replication and drug efficacy in an *ex vivo* model of the central nervous system.

## Introduction

Transmissible spongiform encephalopathies or prion diseases are devastating neurologic diseases of mammals associated with spongiform degeneration and gliosis in the central nervous system (Prusiner, 1991). Prion diseases are caused by unconventional pathogens composed predominately or exclusively of misfolded cellular prion protein (Prusiner, 1982). They replicate by recruiting and converting cellular prion protein (PrP^C^) into disease-associated higher order structures. Prions exist as stable strains that propagate in specific brain areas and even subpopulations of cells in genetically defined rodents (Dickinson and Meikle, 1971; Bruce et al., 1991; Hecker et al., 1992; Bruce, 2003). It is unknown how prions target specific brain regions and lead to distinct disease pathologies (Kimberlin and Walker, 1986).

Rodent models have uncovered essential details on prion strain characteristics. However, the usually long incubation times make bioassays time-consuming and expensive. The recent development of the prion organotypic slice culture assay opens new avenues for studying molecular mechanism of prion propagation *ex vivo* (Falsig et al., 2008). Organotypic slice cultures can be maintained for several months while largely retaining their *in vivo* cytoarchitecture. Importantly, organotypic slice cultures can be easily manipulated chemically (Falsig et al., 2008, 2012) or genetically (Hofmann et al., 2013) and thus represent suitable models to study host cell responses in complex tissue environments. Cerebellar slices efficiently propagate prions and exhibit characteristic features of prion disease such as astro- and microgliosis, loss of dendritic spines, and spongiform changes (Falsig et al., 2008, 2012; Campeau et al., 2013). However, it is unclear if the PrP deposition pattern in infected slices resembles that of *in vivo* infected cerebella.

As the precise molecular nature of the infectious agent is unknown, prions can only be operationally defined. The term PrP^Sc^ often denotes partially proteinase K-resistant PrP. The term PrP^d^ has been used to define disease-associated PrP detected by immunohistochemical analysis of formalin-fixed paraffin embedded sections following antigen retrieval (Jeffrey et al., 2011). Both PrP^Sc^ and PrP^d^ serve as surrogate markers for disease. The use of horseradish peroxidase/chromogenic substrates in immunohistochemistry has major limitations in that detection of multiple antigens in the same section is difficult. Immunostaining using fluorophore-conjugated antibodies has recently been used by the prion community for achieving a higher level of detail on subcellular distribution and simultaneous detection of several antigens within one specimen. Some recent studies successfully employed protein denaturation protocols for the simultaneous detection of disease-associated PrP and cellular markers (Ayers et al., 2011). However, no such staining has been reported for organotypic slice cultures. Instead, histoblot and western blot analysis have been used to study tissue distribution and relative levels of PrP^Sc^ produced upon infection (Falsig et al., 2012). Detailed information as to whether the cellular and subcellular distribution of disease-associated PrP^d^ mimics the *in vivo* situation is missing (Falsig et al., 2008, 2012; Campeau et al., 2013).

We developed a novel staining protocol that combines PrP^d^ denaturation, multicolor immunofluorescence staining, and confocal microscopy of whole mount organotypic cerebellar slices that allows for detailed analysis of cellular and subcellular distribution of abnormal PrP deposits. Here we show that organotypic brain slice cultures recapitulate the neuropathological hallmarks of prion disease and exhibit a PrP^d^ deposition pattern comparable to *in vivo* infected cerebella. The possibility of high resolution microscopy of prion deposits together with the ease of chemical manipulation make organotypic brain slice cultures a highly suitable model for in-depth neuropathological analysis of prion disease progression that limits invasive procedures on living animals.

## Materials and methods

### Ethics statement and mice

Animal care and experimental procedures were conducted according to the institutional animal care committee guidelines and German animal protection laws and were approved by the *Landesamt für Natur, Umwelt, und Verbraucherschutz NRW*. Female C57BL/6JRj mice with pups, at the age of P7, were ordered from Janvier (St. Berthevin Cedex, France) and were housed at a 12:12 light: dark cycle with food and water available *ad libidum*.

### Chemicals

If not otherwise specified, chemicals and reagents were purchased from Sigma (Steinheim, Germany) or Roth (Karlsruhe, Germany). Pefabloc was obtained from Roche (Mannheim, Germany) and staurosporine was purchased from Enzo (Lausen, Switzerland). Ultrapure low-melting-point agarose, slice culture media, supplements and heat inactivated horse serum were obtained from Invitrogen (Darmstadt, Germany). The bicinchoninic acid assay kit was purchased from Pierce (Thermo Scientific, Rockford, USA). The ECL plus chemiluminescence kit was purchased from GE Healthcare (Buckinghamshire, UK). Monoclonal anti-PrP antibody 4H11 has been described previously (Ertmer et al., 2004). The antibody is directed against the globular domain of the protein. Anti-actin antibody was obtained from MP Biomedicals (Eschwede, Germany) and rabbit polyclonal anti-β-3-tubulin antibody was purchased from Covance HISS Diagnostics (Freiburg, Germany). Anti-calbindin D-28K antibody was purchased from Swant (Marly, Switzerland), anti-GFAP antibody was obtained from Dako Cytomation (Hamburg, Germany), anti-iba-1 antibody was purchased from Wako Pure Chemicals Industries (Osaka, Japan), and anti-lamp-1 antibody was obtained from Santa Cruz Biotechnology (Santa Cruz, USA). Fluorescein-conjugated secondary antibodies were purchased from Dianova (Hamburg, Germany) or from Life Technologies (Darmstadt, Germany).

### Preparation of organotypic cerebellar slices and prion infection

Organotypic cerebellar slices were prepared from postnatal day 9–13 (P9–13) old pups of C57BL/6JRj mice according to a previously published protocol (Falsig et al., 2008). Cerebella were extracted and imbedded in 2% liquid low-melting-point agarose. 350 μm thick sections of the medial cerebellum (vermis) were cut with a vibratome (VT1200S, Leica Biosystems, Wetzlar, Germany) and were collected in a reservoir filled with ice cold Gey's balanced salt solution supplemented with 1 mM kynurenic acid and D-(+)-glucose. Agarose and meninges were removed. Prion infection studies were conducted under biosafety containment level two. Mouse-adapted scrapie strain 22L propagated in C57BL/6 mice was used for infection. 10% brain homogenates of healthy control mice and terminally sick mice were prepared according to a standardized protocol in Opti-MEM medium using a dounce homogenizer. Cell debris was pelleted by centrifugation at 872 g, 4°C, for 5 min and the supernatant was stored at –80°C. Up to 10 slices were exposed to 20 mg/ml brain homogenate. Slices were incubated as free-floating sections at 4°C for 1 h under permanent shaking. After three washing steps with cold Gey's balanced salt solution supplemented with 1 mM kynurenic acid and D-(+)-glucose, 2–6 slices were placed on membrane inserts (Millicell-CM inserts, 0.4 μm, 30 mm, Millipore, Carrigtwohill, Co. Cork, Ireland) in 6 well plates containing slice culture medium (100 ml 2 × MEM, 100 ml BME, 100 ml horse serum, 4 ml GlutaMAX-I, 4 ml penicillin/streptomycin, 5.5 ml D-(+)-glucose solution (45%), 86.5 ml H_2_O_bidest_, pH 7.2–7.4). Slices were cultured up to 12 weeks at 37°C, 5% CO_2_ and 95% humidity. Every second day, 95% of the medium was replaced by fresh slice culture medium.

### Dextran sulfate treatment

For the treatment with dextran sulfate (DS-500) before and during the prion infection, cerebellar slices were pretreated for 1 h and were then infected with 22L brain homogenate in the presence of 1–10 μg/ml DS-500. After three rinsing steps, cerebellar slices were cultivated with slice culture medium containing 1–10 μg/ml DS-500 during the first week of cultivation. Cerebellar slices were also treated at later time points during prion infection, starting the third week of cultivation until analysis.

### Propidium iodide staining

Tissue viability was assessed by detection of propidium iodide incorporation into dead cells of cerebellar slices. Cerebellar slices were incubated with 10 μg/ml propidium iodide at 37°C for 2 h. After rinsing the slices with Gey's balanced salt solution supplemented with 1 mM kynurenic acid and D-(+)-glucose, the propidium iodide incorporation was detected by using an inverse fluorescence microscope (Axio observer Z1, Zeiss, Jena, Germany). Slices treated with 5 μM staurosporine were used as positive controls. The exposure time to visually detect the propidium iodide staining was adjusted to the exposure time of slices treated with staurosporine. For comparison, all images were taken using the same microscopic settings.

### Immunofluorescence detection of PrP^d^ by epifluorescence and confocal microscopy

Whole mount cerebellar slices were rinsed with PBS, fixed with 4% PFA at RT for 2 h, and permeabilized with 0.5% Triton X-100 at 4°C for 18 h. PrP was denatured with 6 M guanidine hydrochloride or >98% formic acid at RT for 3 h for demasking epitopes in PrP^d^. After rinsing, cerebellar slices were blocked with 5% BSA up to 3 days and slices were incubated with the primary antibodies diluted in 5% BSA at 4°C for 3 days, followed by extensive washing and incubation with the suitable fluorophore-conjugated secondary antibodies diluted in 5% BSA at 4°C for 3 days. Cellular nuclei were stained with Hoechst (Molecular Probes, Eugene, Oregon, USA) using a 1:10,000 dilution in PBS at RT. The analysis of the samples was conducted with an inverse epifluorescence microscope (Axio observer Z1, Zeiss, Jena, Germany) or with an automated epifluorescence microscope (Axio scan Z1, Zeiss, Jena, Germany) for an overview using the tile scanning function. For high-resolution images a LSM 700 laser scanning microscope (Zeiss, Jena, Germany) was used. Images were taken in a focal plane ~8 μm below the top of the slice. To quantify differences between fluorophore intensities, channel settings were kept constant within one experiment or between various experiments. Mock exposed slices were included in every experiment and were used as controls for residual background staining of PrP^C^.

### Western blot analysis

Two slices were pooled and lysed in lysis buffer (0.5% deoxycholic acid sodium salt, 0.5% Nonidet P40 substitute). Lysis of the tissue was performed by three freeze and thaw cycles, including dissociation of the tissue using a pipette and sonication (Sonoplus HD3200, Bandelin Sonorex Technik, Berlin, Germany). If needed, a short centrifugation step was included. Precipitates were cleared by a low speed centrifugation (1153 g, 4°C, 3 min). The protein concentration of the lysates was determined using the bicinchoninic acid assay kit (Thermo Scientific, Rockford, USA). For the specific detection of PrP^Sc^, 20 μg protein was digested with 62.5 μg/ml proteinase K at 37°C for 30 min. Proteolysis was terminated by adding Pefabloc SC and NuPAGE LDS sample buffer (Invitrogen, Darmstadt, Germany) and by boiling the sample at 95°C for 5 min. 5–10 μg protein was used to assay for actin as a loading control. Proteins were separated on NuPAGE Novex 4–12% Bis-Tris Midi gels (Invitrogen, Darmstadt, Germany) and were detected by western blot using primary antibodies and horseradish peroxidase-conjugated secondary antibodies.

### Statistics

Data sets were analyzed with the One-way analysis of variances (ANOVA). To evaluate statistically significant differences between control and sample, Dunnett's or Tukey's multiple comparisons *post-hoc* test or unpaired two-tailed *t*-test were used. *P*-values less than 0.05 were considered significant (^*^p < 0.05, ^**^p < 0.01, ^***^p < 0.001, ^****^p < 0.0001). Error bars represent standard deviation (SD) and the sample size (*n*) was at least three or more.

## Results

### Propagation of mouse-adapted prion strain 22L in C57BL/6JRj cerebellar slices

**Figure 1:**
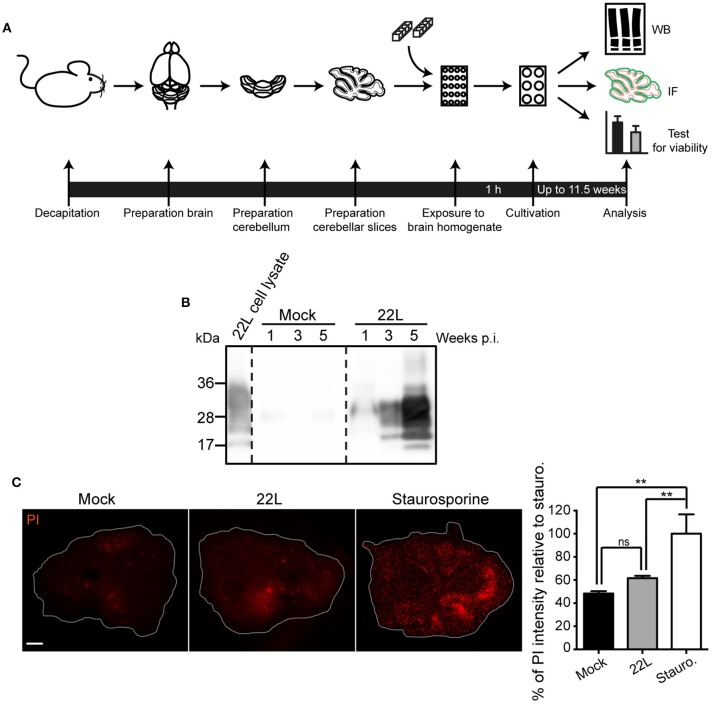
**Viability of sagittal cerebellar slices infected with 22L prions. (A)** Experimental setup. Organotypic slices of cerebella from 9 to 13 days old C57BL/6JRj mice were prepared and exposed to 20 mg/ml 22L brain homogenate or Mock brain homogenate. Slices were cultured on membrane inserts for up to 11.5 weeks. **(B)** Cerebellar slices exposed to Mock brain homogenate or infected with 22L prions were cultured for up to 5 weeks and slice lysates were subjected to proteinase K (PK) treatment. PrP^Sc^ was detected by western blot using mAb 4H11. **(C)** Slices were cultured for 10.5 weeks and subsequently stained for dead cells using propidium iodide (PI). Slices treated with 5 μM staurosporine served as positive controls. Samples were analyzed by epifluorescence microscopy using the tile scanning function with identical imaging settings. Scale bar: 500 μm. Statistical analysis was performed using One-way ANOVA with Tukey's multiple comparisons test. Asterisks indicate significant changes (*n* = 3; ^**^p < 0.01; ns, not significant). WB, western blot; IF, immunofluorescence; p.i., post infection; Stauro., staurosporine.

We chose wildtype C57BL/6JRj mice for preparation of cerebellar slices to monitor prion infection under normal cerebellar PrP^C^ expression conditions. Sagittal 350 μm sections cut through the cerebellar vermis of pups at postnatal day 9–13 (P9–13) were exposed to normal brain homogenate or infected with scrapie brain homogenate and slices were subsequently cultured for a period of up to 11.5 weeks (Figure 1A). Western blot analysis of proteinase K (PK) resistant PrP (PrP^Sc^) at different time points post infection (p.i.) revealed a dramatic increase of PrP^Sc^ over time, demonstrating an efficient propagation of prion strain 22L. No PrP^Sc^ was detected in Mock exposed slices (Figure 1B). Prion infection did not significantly decrease cell viability compared to Mock exposed slices at 10.5 weeks post exposure to prions (Figure 1C). In conclusion, prion infected and Mock exposed slices are viable over a cultivation period of more than 10 weeks.

### Detection of disease-associated PrP^d^ following antigen denaturation

**Figure 2:**
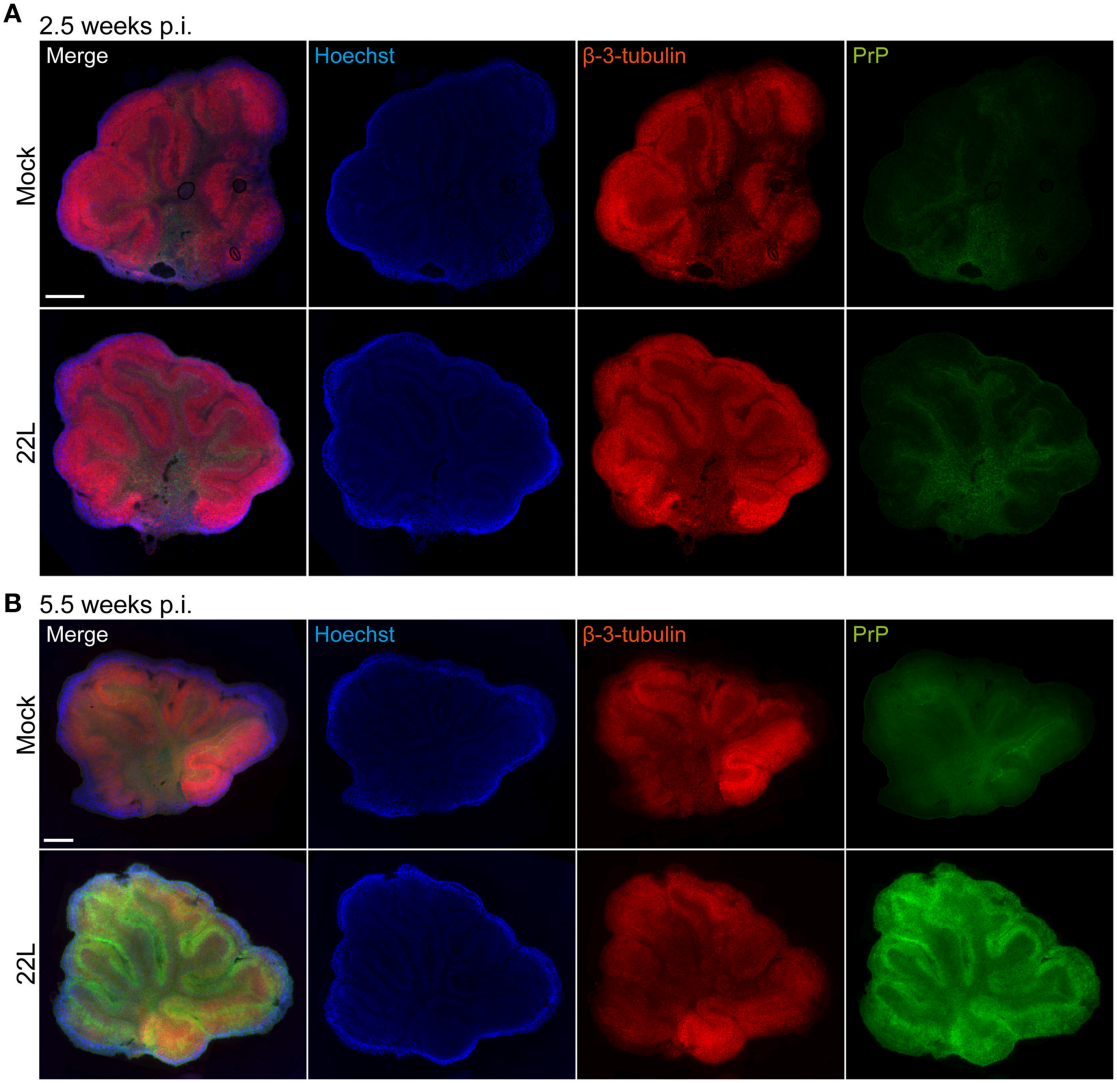
**Immunofluorescence detection of PrP^d^ in cerebellar slices treated with guanidine hydrochloride 5.5 weeks post exposure to 22L prions**. Cerebellar slices were exposed to Mock brain homogenate or infected with 22L prions and cultured for **(A)** 2.5 or **(B)** 5.5 weeks. Slices were treated with guanidine hydrochloride for the specific detection of PrP^d^ with mAb 4H11 (green). “PrP” and “β-3-tubulin” in the top left corner of the images denote the antigens detected by the specific antibodies. Slight background staining for PrP was observed in Mock exposed slices by epifluorescence microscopy of whole slices. Neurons were stained with pAb against β-3-tubulin (red), nuclei were counterstained with Hoechst. All samples were analyzed by epifluorescence microscopy using the tile scanning function with identical imaging settings for each sample group. Scale bar: 500 μm.

Propagation of prions in organotypic slices has previously been assessed by western blot or histoblot techniques (Falsig et al., 2012). These techniques provide only limited information on the cellular distribution of disease-associated prion protein in the slice. We developed an epitope denaturation protocol to visualize the cellular localization of disease-associated PrP and cellular markers in organotypic brain slices. We use the term PrP^d^ to refer to disease-associated PrP detected by fluorophore-conjugated antibodies following antigen denaturation. PrP^d^ detected by immunohistochemistry likely comprises both proteinase K sensitive and resistant forms (Jeffrey et al., 2011). We chose slices 5.5 weeks post exposure, a time point at which prominent PrP^Sc^ accumulation was detected by western blot analysis (Figure 1B). Several protocols for antigen denaturation were compared using prion infected and Mock exposed slices, varying denaturation methods and incubation times (Figure 2, Supplementary Figure 1). Whole mount slices were stained without reslicing. Continuous culture led to thinning of the slices to approximately 30 μm. Epifluorescence microscopy using tile scanning function and identical imaging settings for each sample group revealed the stereotypical vermis foliation pattern consisting of 10 lobules. The tissue structure with granular layer, Purkinje cell layer, and molecular layer was grossly preserved. No difference in PrP distribution was observed between 5.5 week old Mock exposed and 22L infected slices when antigen denaturation was omitted (Supplementary Figure 1A). Antigen denaturation with formic acid for 3 h slightly enhanced PrP detection in prion infected slices, but relatively high PrP signals were apparent also in Mock exposed slices. Hoechst staining appeared slightly blurred by formic acid treatment (Supplementary Figure 1B). Varying the incubation times for the formic acid did not improve the staining results for Hoechst or PrP^d^ (data not shown). Best results were obtained upon antigen denaturation by 6 M guanidine hydrochloride (Figure 2; Taraboulos et al., 1990). Control experiments using only fluorophor-conjugated secondary antibodies or combinations of primary antibodies with three species-specific secondary antibodies did not reveal any background staining or cross-reactivity of antibodies (data not shown). Consistent with our western blot data (Figure 1B), no distinct staining of PrP^d^ was detected in 22L infected slices compared to Mock exposed slices 2.5 weeks p.i. (Figure 2A). At 5.5 weeks p.i., a strong increase in PrP^d^ immunoreactivity was observed over a slight PrP background in Mock exposed slices (Figure 2B). Guanidine hydrochloride treatment had no discernible effect on the immunoreactivity of β-3-tubulin or nuclear staining using Hoechst.

### Localization of PrP^d^ in molecular and Purkinje cell layers

**Figure 3:**
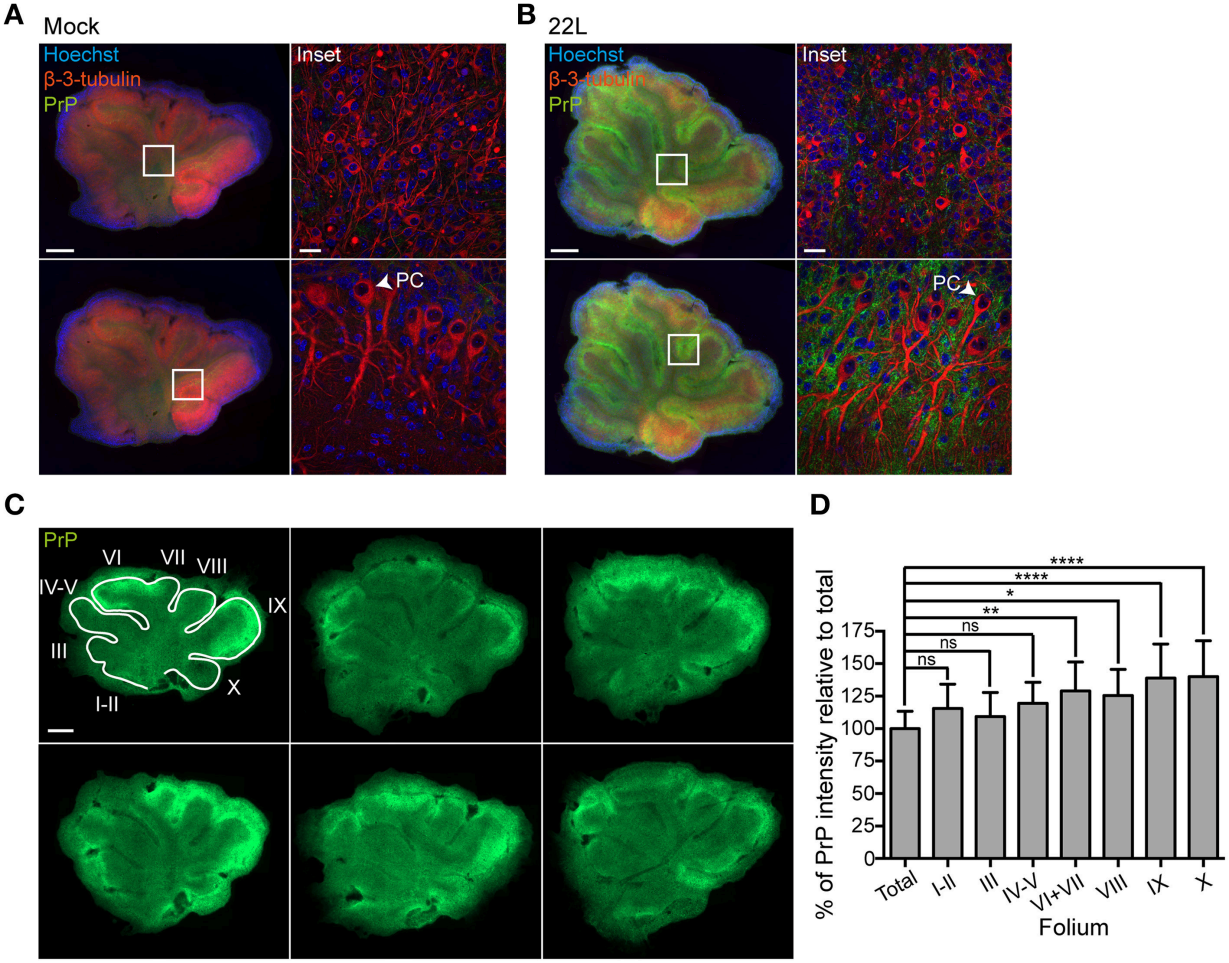
**PrP^d^ levels in molecular and Purkinje cell layers. (A,B)** Cerebellar slices exposed to Mock brain homogenate or infected with 22L prions and cultured for 5.5 weeks were stained for PrP^d^ (green) following antigen denaturation. PAb against β-3-tubulin (red) was used to label neurons, nuclei were counterstained with Hoechst. Samples were analyzed by epifluorescence microscopy using the tile scanning function (left panels, scale bar: 500 μm). Insets of the granular layer including the white matter (upper panels) and the molecular layer including the Purkinje cell layer (PC) (lower panels) were analyzed by confocal microscopy (right panels, scale bar: 20 μm). **(C)** Epifluorescence images of cerebellar slices 9 weeks p.i. stained for PrP^d^ (green) following antigen denaturation. Scale bar: 500 μm. **(D)** Cerebellar folia I-X (from anterior to posterior) were manually demarcated and the PrP intensity per folium was quantified relative to the PrP intensity of the whole slice set to 100% (total). Bars represent mean values ±SD of 14 slices. Statistical analysis was performed using One-way ANOVA with Dunnett's multiple comparisons test. Asterisks indicate significant changes (^****^p < 0.0001; ^**^p < 0.01; ^*^p < 0.05; ns, not significant).

PrP^d^ deposition patterns were assessed by confocal microscopy in slices 5.5 weeks post exposure to brain homogenates (Figures 3A,B). Only moderate PrP^d^ staining was detected in the granular layer (upper panels) and the white matter of cerebellar slices infected with 22L prions. By contrast, widespread PrP^d^ deposition was observed in the neuropil of the molecular and Purkinje cell layers (Figure 3B, lower panels). Purkinje cell layers (Purkinje cells are marked with arrowheads) of both prion infected and Mock exposed slices were arranged as a multilayer, consistent with the reported broadening of cell layers *ex vivo* (Falsig et al., 2008). Amyloid plaques were absent from prion or Mock infected slices. Consistent with *in vivo* studies, Purkinje cells did not exhibit PrP^d^ staining (Moore et al., 2008; Sarasa et al., 2012).

The mammalian cerebellum is subdivided into several lobes separated by fissures. Most prominent fissures compartmentalize the cerebellum into anterior lobe (anterior folia I–V), middle lobe (folia VI–VII), and caudal lobe (folia VIII–X) (Herrup and Kuemerle, 1997). Epifluorescence analysis revealed that folia differed in PrP^d^ deposition patterns, with folia VI-X generally exhibiting stronger stainings compared to folia I-V. Quantitative analysis of PrP fluorescence intensities of whole slices 9 weeks post exposure (Figure 3C) confirmed signifcantly higher PrP^d^ intensities in folia VI + VII, VIII, IX, and X (Figure 3D).

### Neuropathological changes in 22L infected cerebellar slices

**Figure 4:**
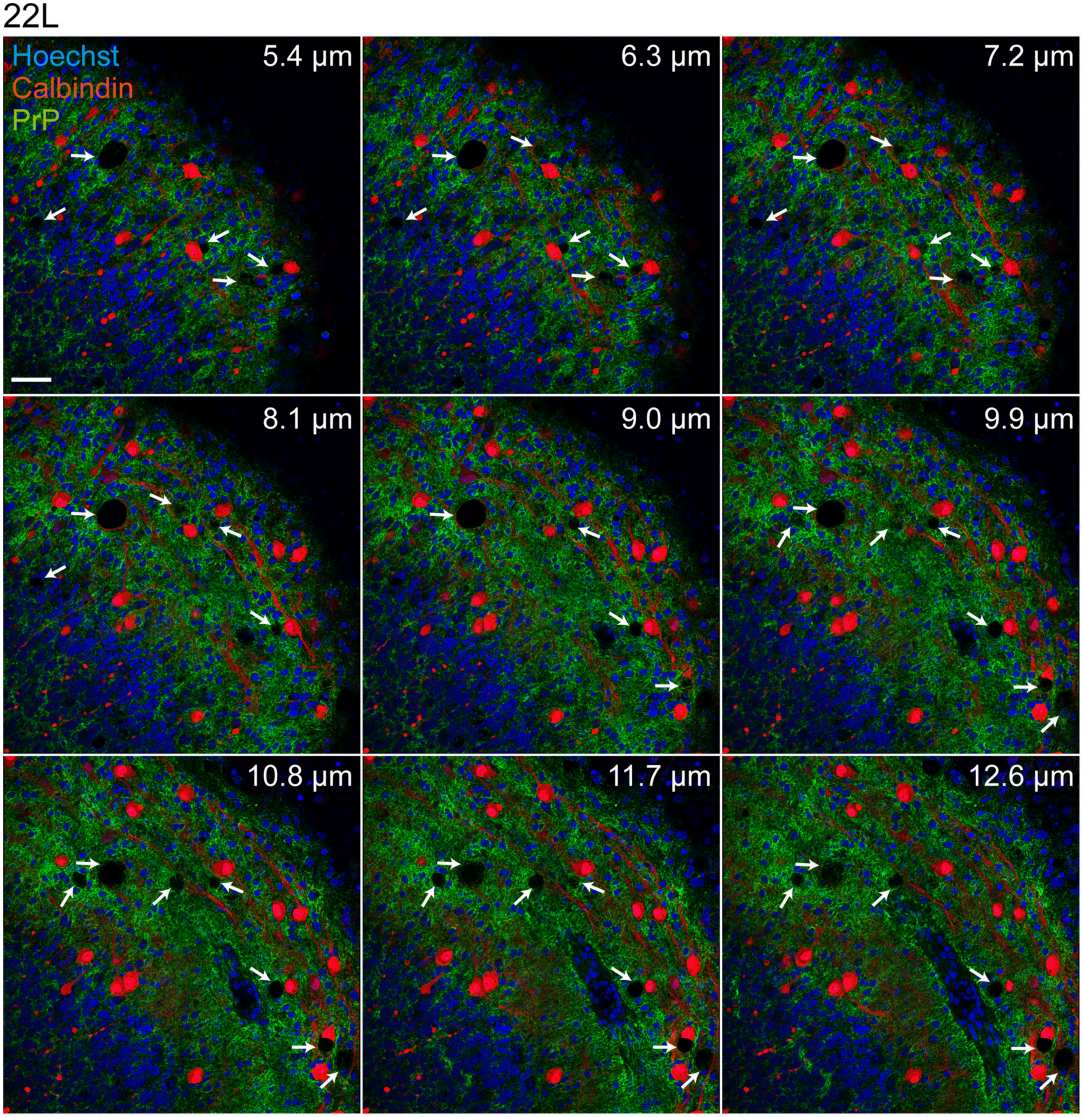
**Spongiform changes in cerebellar slices 9 weeks post exposure to 22L prions**. Following antigen denaturation, PrP^d^ was detected with mAb 4H11 (green), Purkinje cells were detected using anti-calbindin mAb (red). Nuclei were counterstained with Hoechst. Cerebellar slices exposed to Mock brain homogenate served as controls (data not shown). Samples were analyzed by confocal microscopy using the tile scanning and z-stack functions with identical imaging settings. Consecutive sections through the slice were imaged every 0.9 μm from the bottom to the top. Shown are nine consecutive sections from the z-layer of the slices. Arrows mark vacuolations. Scale bar: 50 μm.

**Figure 5:**
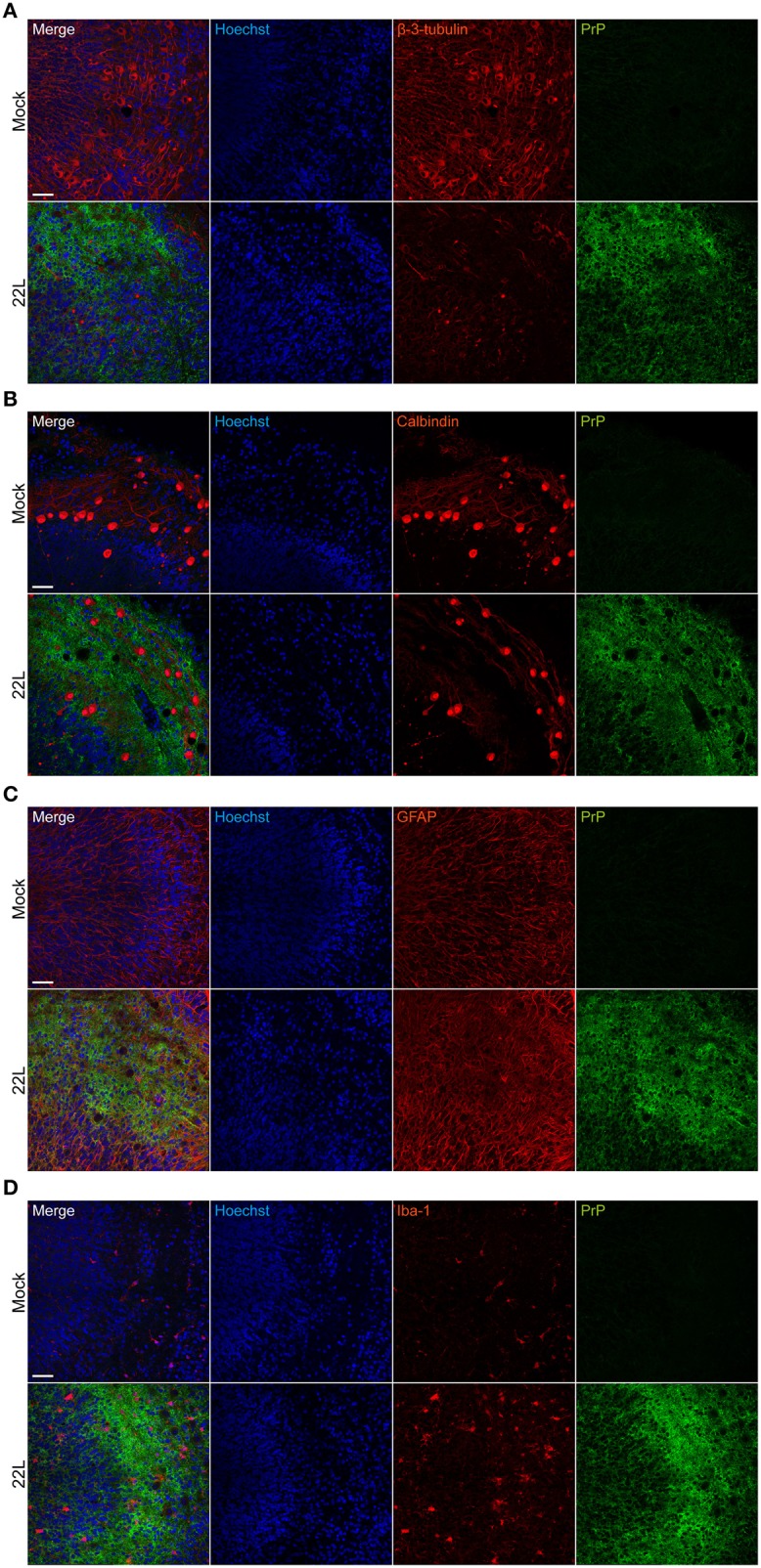
**Neuropathological changes in 22L infected cerebellar slices**. Slices exposed to Mock or 22L brain homogenate were cultured for 9 weeks. PrP^d^ was detected following antigen denaturation using mAb 4H11 (green). **(A)** Neurons were stained with pAb anti-β-3-tubulin (red). **(B)** Purkinje cells were labeled with mAb against calbindin (red). **(C)** PAb anti-GFAP was used to stain astrocytes (red). **(D)** Microglia were detected with pAb anti-iba-1 (red). Nuclei were counterstained with Hoechst. Molecular and Purkinje cell layers of folia IX were imaged by confocal microscopy using the tile scanning function with identical imaging settings for each sample group. Scale bar: 50 μm.

Consecutive sections of 22L infected and Mock exposed slices 9 weeks p.i. were imaged for vacuole formation (Figure 4). Consistent with *in vivo* vacuolization patterns (Kim et al., 1990a), spongiform changes were also apparent in the gray matter, particularly in molecular and Purkinje cell layers of 22L infected organotypic cerebellar slices. No vacuolation was observed in Mock exposed slices imaged using the same settings (data not shown). Confocal immunofluorescence analysis of 22L infected slices 9 weeks p.i. (~10 μm from the top of the slice) demonstrated a substantial reduction in β-3-tubulin immunoreactivity compared to Mock exposed slices, indicative of neuronal loss (Figure 5A). Numbers of Purkinje cells appeared reduced upon prion infection (Figure 5B). Purkinje cells in Mock exposed slices exhibited a well-defined arborization penetrating into the molecular layer of the cerebellum. By contrast, arborizations of Purkinje cells in 22L infected slices appeared degenerated. An increase in GFAP immunoreactivity was observed in prion infected compared to Mock exposed slices (Figure 5C). Concomitantly, the number of iba-1 positive microglia increased in 22L infected slices (Figure 5D). In summary, characteristic hallmarks of prion diseases including spongiform changes, Purkinje cell degeneration, astro- and microgliosis were reproduced in 22L infected organotypic cerebellar slices from C57BL/6JRj mice.

### PrP^d^ localizes in the neuropil associated with astrocytes and microglia

**Figure 6:**
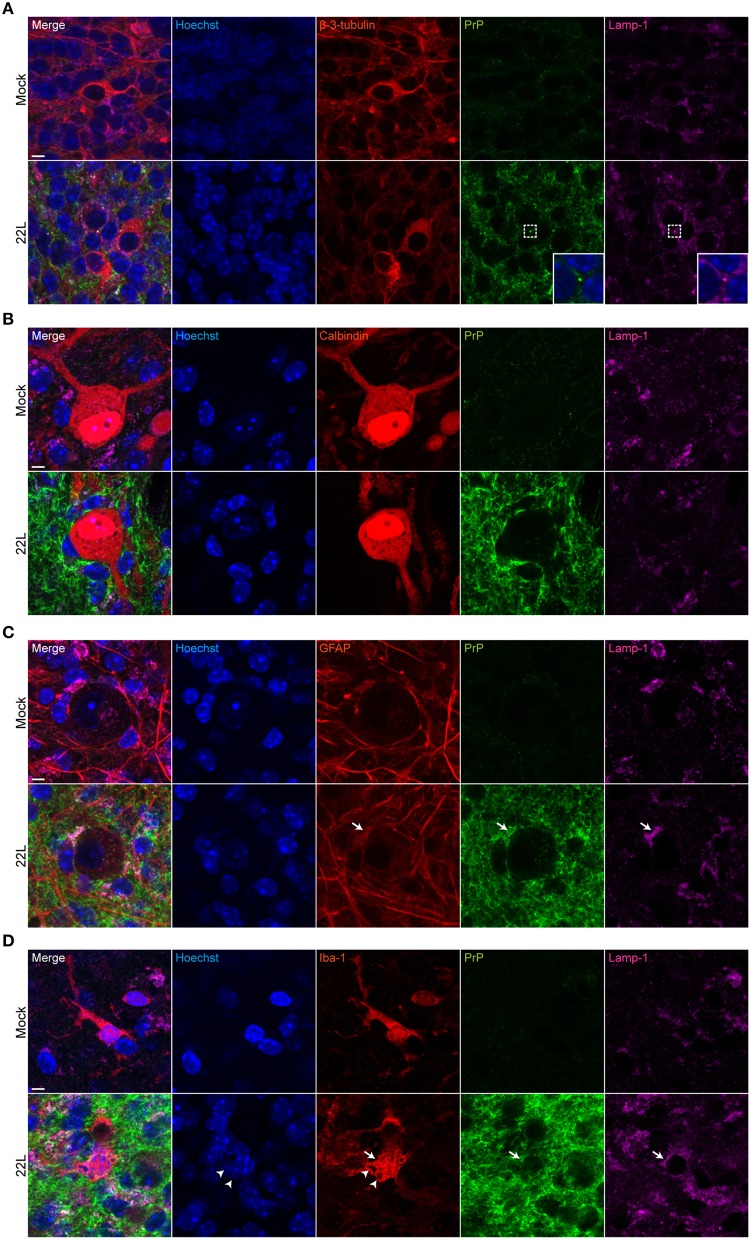
**Cellular localization of PrP^d^ in cerebellar slices**. 9 weeks p.i., Mock exposed or 22L infected slices were subjected to antigen denaturation and immunofluorescence staining. Cells in the molecular layer of folium IX were analyzed by confocal microscopy using digital zoom with identical imaging settings for each sample group. PrP^d^ was detected with mAb 4H11 (green). **(A)** Neurons were stained for β-3-tubulin (red). The insets respresent magnifications of the indicated region. **(B)** Purkinje cells were stained with mAb anti-calbindin (red). **(C)** Astrocytes were labeled with pAb anti-GFAP (red). Arrows mark co-localization of PrP^d^ with lamp-1 within astrocytes. **(D)** PAb anti-iba-1 was used to detect microglia (red). Arrowheads mark degenerated nuclei within microglia, arrows mark lysosomes. Lysosomes were labeled with mAb anti-lamp-1 (purple) and nuclei were counterstained with Hoechst. Mock exposed slices served as controls. Scale bar: 5 μm.

To identify the cellular localization of PrP^d^, double labeling of PrP^d^ with neurons (β-3-tubulin), Purkinje cells (calbindin), astrocytes (GFAP), and microglia (iba-1) was performed. Potential subcellular deposition of PrP^d^ within lysosomes was assessed using mAb targeting lamp-1. The molecular and Purkinje cell layers exhibited intense spot-like to fibrillar PrP^d^ deposits (Figure 6). Spot-like PrP^d^ deposition was observed in areas with β-3-tubulin staining (Figure 6A). In some cases, PrP^d^ colocalized with neuronal lysosomes (Figure 6A, insets). Purkinje cells were surrounded by cells with intense PrP^d^ staining, while Purkinje cell bodies showed no or only very faint staining for PrP^d^ (Figure 6B; Supplementary Figure 2). Interestingly, PrP^d^ deposition was found extracellularly between Purkinje cells in close vicinity (Supplementary Figure 2). Surrounding cells stained positive for glial fibrillar acidic protein (GFAP), suggesting that they represent Bergmann glia (Figure 6C). Here, PrP^d^ at least partially colocalized with lamp-1, indicative of lysosomal localization of PrP^d^ within astrocytes (Figure 6C, arrows). PrP^d^ deposition in astrocytes has been reported previously for 22L infected C57BL/6 mice (Diedrich et al., 1991). PrP^d^ staining was also associated with microglia (Figure 6D). In some instances, degenerated nuclei were identified in iba-1 positive microglia in the 22L infected cerebellar slices (Figure 6D, arrowheads). In conclusion, in 22L infected cerebellar slices from C57BL/6JRj mice, PrP^d^ deposits as spot-like or fibrillar structures in the neuropil and is associated with astrocytes, microglia and neurons. In line with *in vivo* studies (Moore et al., 2008; Sarasa et al., 2012), PrP^d^ is not (or only at very low levels) located in Purkinje cells.

### Anti-prion compound dextran sulfate reduces abnormal prion protein deposition

**Figure 7:**
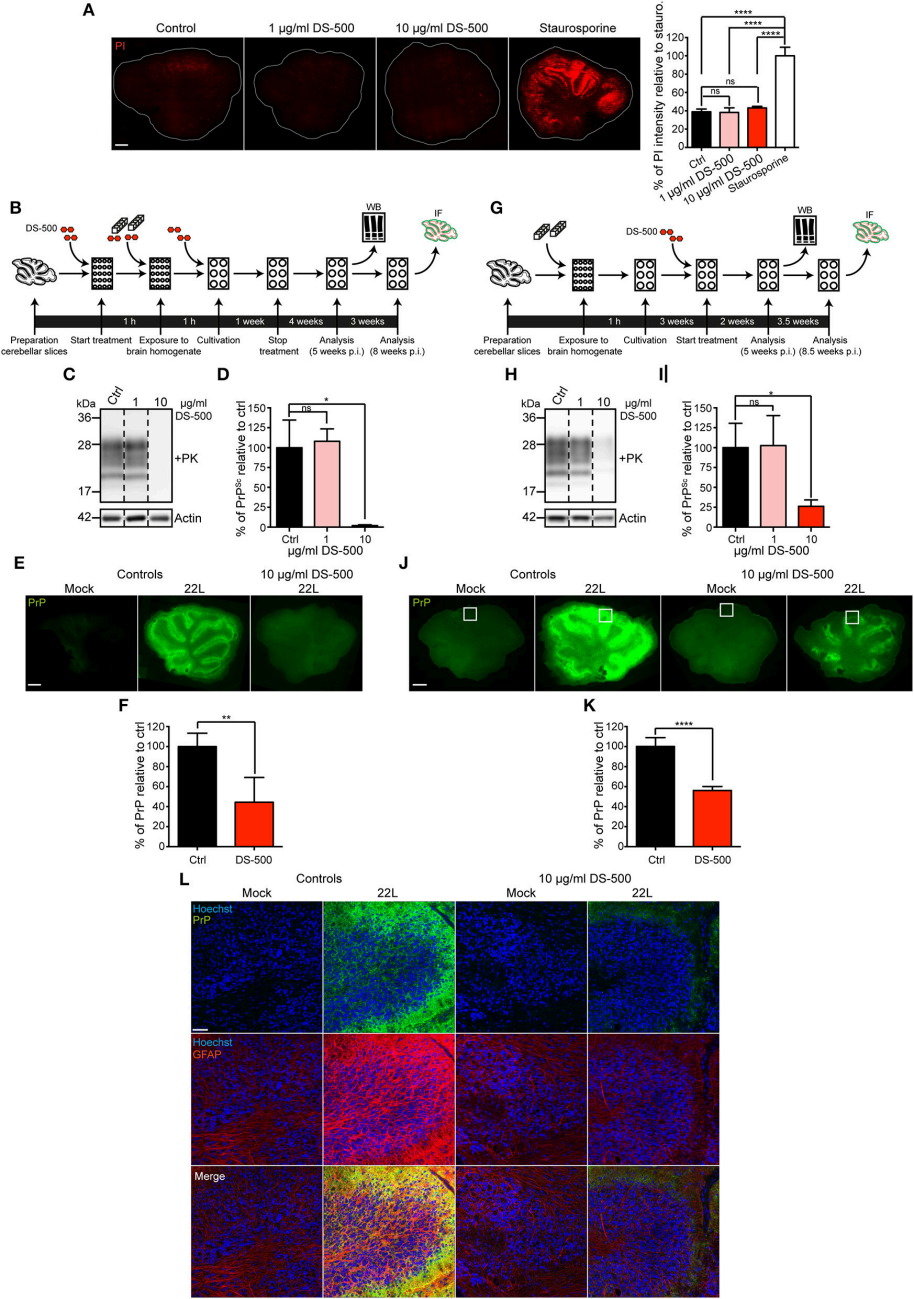
**Anti-prion compound DS-500 reduces abnormal prion protein deposition. (A)** Viability of slices cultured in the presence or absence of different concentrations of DS-500. Slices were cultured for 1 week, then 1–10 μg/ml DS-500 were added for 5 weeks. Slices were subsequently stained for dead cells using propidium iodide (PI). Slices treated with 5 μM staurosporine served as positive controls. Samples were analyzed by epifluorescence microscopy using the tile scanning function with identical imaging settings. Scale bar: 500 μm. Statistical analysis was performed using One-way ANOVA with Tukey's multiple comparisons test. Asterisks indicate significant changes (*n* = 3; ^****^p < 0.0001; ns, not significant). **(B)** Experimental setup to study the effect of DS-500 on the establishment of a 22L prion infection in cerebellar slices. Cerebellar slices were pretreated with different concentrations of DS-500 for 1 h and exposed to inoculum in the presence of DS-500 for 1 h. Slices were maintained in medium containing DS-500 for 1 week. Slices were kept in normal medium for up to 8 weeks p.i. **(C)** Cerebellar slices were lysed at week 5 p.i. and lysates were proteinase K-digested. MAb 4H11 was used to analyze PK-resistant PrP (PrP^Sc^) by western blot. Actin was detected as a loading control in –PK samples. Mock treated slices served as controls. For presentation purposes additional lanes were excised. **(D)** Statistical analysis of PrP^Sc^ levels detected by western blot. Bars represent mean values ± SD (*n* = 5). Statistical analysis was performed using One-way ANOVA with Dunnett's multiple comparisons test. Asterisks indicate significant changes in the mean values of DS-500 treated samples relative to control samples set to 100% (^*^p < 0.05; ns, not significant). **(E)** Specific detection of PrP^d^ following antigen denaturation (green). Samples were analyzed by epifluorescence microscopy using the tile scanning function with identical imaging settings. Scale bar: 500 μm. **(F)** PrP^d^ intensity of 22L infected slices treated with 10 μg/ml DS-500 was quantified relative to 22L infected control slices set to 100%. Bars represent mean values ± SD (*n* = 4). Statistical analysis was performed using unpaired two-tailed *t*-test (^**^p < 0.01; ns, not significant). **(G)** Experimental setup to study the effect of DS-500 on persistent 22L prion infection in cerebellar slices. After exposure to inoculum for 1 h, slices were cultured with normal medium for 3 weeks. Subsequently, slices were cultured in the presence of DS-500 until 5 and 8.5 weeks p.i. **(H)** PK-resistant PrP (PrP^Sc^) present in slices 5 weeks p.i. was detected by western blot using mAb 4H11. Actin was detected in -PK samples as loading control. For presentation purposes additional lanes were excised. No statistically significant changes in actin levels were observed between infected and uninfected slices (data not shown). **(I)** Quantitative comparison of PrP^Sc^ levels in prion-infected control slices and DS-500 treated, infected slices. The amount of PrP^Sc^ in the 22L infected control slices was set to 100% and was compared to the amount of PrP^Sc^ in the DS-500 treated, infected slices. Bars represent mean values ± SD (*n* = 3; ^*^p < 0.05; ns, not significant). **(J)** Detection of PrP^d^ 8.5 weeks p.i. following antigen denaturation. Samples were analyzed by epifluorescence microscopy using tile scanning function with identical imaging settings. Scale bar: 500 μm. Shown are prion infected and Mock exposed slices that were/were not treated with 10 μg/ml DS-500 for 5.5 weeks. The boxed region was chosen for confocal microscopy analysis in **(L). (K)** PrP^d^ intensity of 22L infected slices treated with 10 μg/ml DS-500 for 5.5 weeks was quantified relative to 22L infected control slices set to 100%. Bars represent mean values ± SD (*n* > 4, ^****^p < 0.0001). **(L)** Molecular and Purkinje cell layers of folia VII (boxed area in **J**) were imaged by confocal microscopy using the tile scanning function with identical imaging settings for each sample group. PAb anti-GFAP was used to stain astrocytes (red). PrP^d^ was detected with mAb 4H11 (green). Nuclei were counterstained with Hoechst. Scale bar: 50 μm. WB, western blot; IF, immunofluorescence.

To further confirm that the PrP staining observed in prion infected slices correlates with prion infection, we treated prion infected and uninfected slices with a known anti-prion compound and monitored PrP^d^ staining. Previous studies have demonstrated that anti-prion compounds can inhibit chronic prion infections also in cerebellar slices (Falsig et al., 2012). DS-500 is a sulfated polysaccharide that has been used extensively as a glycosaminoglycan mimetic with anti-prion activity *in vivo* and in cell cultures (Ehlers and Diringer, 1984; Caughey and Raymond, 1993; Wolf et al., 2015). The presence of up to 10 μg/ml DS-500 in the culture medium over a period of 5 weeks had no significant effect on the viability of brain slices (Figure 7A). DS-500 was added to the slices either during the infection period (Figure 7B) or 3 weeks p.i. (Figure 7G). Treatment of slices with 10 μg/ml DS-500 during the first week of infection was sufficient to drastically reduce prion replication (Figures 7C–F). Slices that were treated with 10 μg/ml DS-500 during the first week of infection showed significantly reduced levels of PrP^Sc^ as assessed by western blot (Figures 7C,D) and PrP^d^ detected by immunofluorescence staining of whole mount slices (Figures 7E,F). DS-500 also significantly reduced PrP^Sc^ and PrP^d^ when added for up to 5.5 weeks to slices with ongoing prion infection (Figures 7H–K). The fact that the reduction of PrP^Sc^ correlated with the reduction of PrP^d^ (Figure 7L) strongly argues that our staining method indeed detects disease-associated PrP.

We further assessed if continuous DS-500 treatment of prion infected slices could reduce astrocytosis. Intense astrocytosis was detected in 22L infected slices 8.5 weeks p.i. to prions (Figure 7L). By contrast, in slices with ongoing DS-500 treatment (10 μg/ml), GFAP staining was comparable to Mock exposed slices. In summary, the antigen denaturation technique used here allows detailed analysis of disease-associated prion protein deposition and pathologic changes upon prion infection *ex vivo*.

## Discussion

Organotypic slices represent a suitable alternative to *in vivo* experiments and allow the analysis of prion replication in a complex cell environment within weeks post infection (Falsig et al., 2012). A limitation of this model so far was that the cellular and subcellular distribution of disease-associated PrP could not be assessed. Here we report the development of an antigen denaturation protocol suitable for the simultaneous detection of abnormal prion protein deposition and cellular markers in organotypic slices. The combination of a prion susceptible *ex vivo* central nervous system model and the simultaneous detection of disease-specific PrP and cellular markers opens the avenue for detailed analysis of prion replication and new modes of intervention.

We studied in detail the pathologic consequences of infection of wildtype organotypic cerebellar slices with mouse-adapted prion strain 22L that specifically targets the cerebellum (Karapetyan et al., 2009). In cerebellar wildtype slices, prion strain 22L induced characteristic neuropathological changes approximately 8–9 weeks post exposure to prions. Interestingly, no significant changes in propidium iodide uptake were identified between infected and uninfected slices, arguing that slices were still viable. PrP^Sc^ propagation was readily detected by western blot as soon as 21 days post infection with 22L, comparable to the infection period required for PrP^Sc^ detection using slices from transgenic tga20 mice that show tenfold overexpression of mouse PrP (Falsig et al., 2008). This finding is in agreement with *in vivo* data that demonstrate that the higher cerebellar PrP^C^ expression in this mouse model does not correlate with increased PrP^Sc^ accumulation (Fischer et al., 1996; Karapetyan et al., 2009).

The 22L PrP^d^ deposition pattern identified in *ex vivo* infected C57BL/6 cerebellar slices closely resembled that of *in vivo* infected cerebella, strongly arguing that the 22L strain-specific cell tropism is retained *ex vivo*. PrP^d^ deposition appeared granular and was most prominent in molecular and Purkinje cell layers. Purkinje cell bodies were usually negative for PrP^d^, with no or only very faint PrP^d^ signals detected. Although not primary targets of prion replication (Kim et al., 1990b), Purkinje cells appeared reduced in numbers and degenerated. The reason for Purkinje cell degeneration is unclear but might be due to extrinsic causes, such as loss of granule neurons or dysfunction of glia. PrP^d^ staining appeared sometimes confined to extracellular spaces (Supplementary Figure 2). In some instances, PrP^d^ was found to colocalize with lysosomal marker lamp-1 within neurons, GFAP-positive cells and microglia. As microglia are non-permissive to prion infection (Priller et al., 2006), the presence of PrP^d^ in microglia might be due to uptake of PrP^d^ or infected cells undergoing cell death. Consistent with pathologic changes associated with 22L replication *in vivo* (Kim et al., 1990a,b; Williams et al., 1997), we observed a severely diminished number of granule neurons in prion infected slices. Furthermore, reactive astro- and microgliosis was a prominent feature in areas with abundant PrP^d^ deposition.

In this study we have also shown that the effect of anti-prion drugs can be tested both during acute and chronic prion infections. We have used DS-500, a sulfated polyanion with known anti-prion activity (Ehlers and Diringer, 1984; Caughey and Raymond, 1993; Wolf et al., 2015). The mode of action of DS-500 and other glycosaminoglycan mimetics is complex and likely involves direct effects on PrP^Sc^ synthesis and on PrP^Sc^ stabilization. DS-500 has limited ability to cross the blood brain barrier and thus likely impairs peripheral prion replication (Koster et al., 2003). The experiments presented here demonstrate that DS-500 also exhibits potent anti-prion activity in the central nervous system. How exactly DS-500 impairs prion replication is unknown. Our recent study in several cell culture models argues, that prion entry into target cells is not impaired by DS-500 (Wolf et al., 2015). It is thus likely that DS-500 counteracts prion infection downstream of prion uptake.

Many pharmaceuticals are incapable of crossing the blood brain barrier (Pardridge, 2012). While their use as therapeutics for prion diseases is limited, testing the anti-prion efficacy of drugs with known targets in organotypic brain slices will help to understand the molecular mechanisms underlying prion propagation in the central nervous system.

## Funding

This work was supported by the Helmholtz Association HAI-NDR grant SO-083, APRI Exploration grant “Cell tropism and prion strains” and DFG grant VO 1277/1-3.

### Conflict of interest statement

The authors declare that the research was conducted in the absence of any commercial or financial relationships that could be construed as a potential conflict of interest.

## Abbreviations

DS-500: Dextran sulfate 500
GFAP: Glial fibrillary acidic protein
p.i.: post infection
PrP^C^: Prion Protein
PrP^Sc^: Proteinase K resistant PrP
PrP^d^, Disease-associated PrP: detected by immunofluorescence following guanidine hydrochloride treatment
PK: Proteinase K.
