# Supplementary material for: Deposition pattern and subcellular distribution of disease-associated prion protein in cerebellar organotypic slice cultures infected with scrapie
Source: Front Neurosci. 2015 Nov 4;9:410. doi: 10.3389/fnins.2015.00410 (PMC4631830; doi:10.3389/fnins.2015.00410)
Supplement: Supplementary file 1 [file DataSheet1.DOCX]

***Supplementary Material***

**Deposition pattern and subcellular distribution of disease-associated prion protein in cerebellar organotypic slice cultures infected with scrapie**

**Hanna Wolf^1^, André Hossinger^1^, Andrea Fehlinger^1^, Sven Büttner^1^, Valerie Sim^2^, Debbie McKenzie^2^ and Ina Vorberg^1, 3, *^**

*** Correspondence:** Prof. Dr. Ina Vorberg, Deutsches Zentrum für Neurodegenerative Erkrankungen e.V., Ludwig-Erhard-Allee 2, 53175 Bonn, Germany, (Email) [ina.vorberg@dzne.de](mailto:ina.vorberg@dzne.de)

1. **Supplementary Figures**


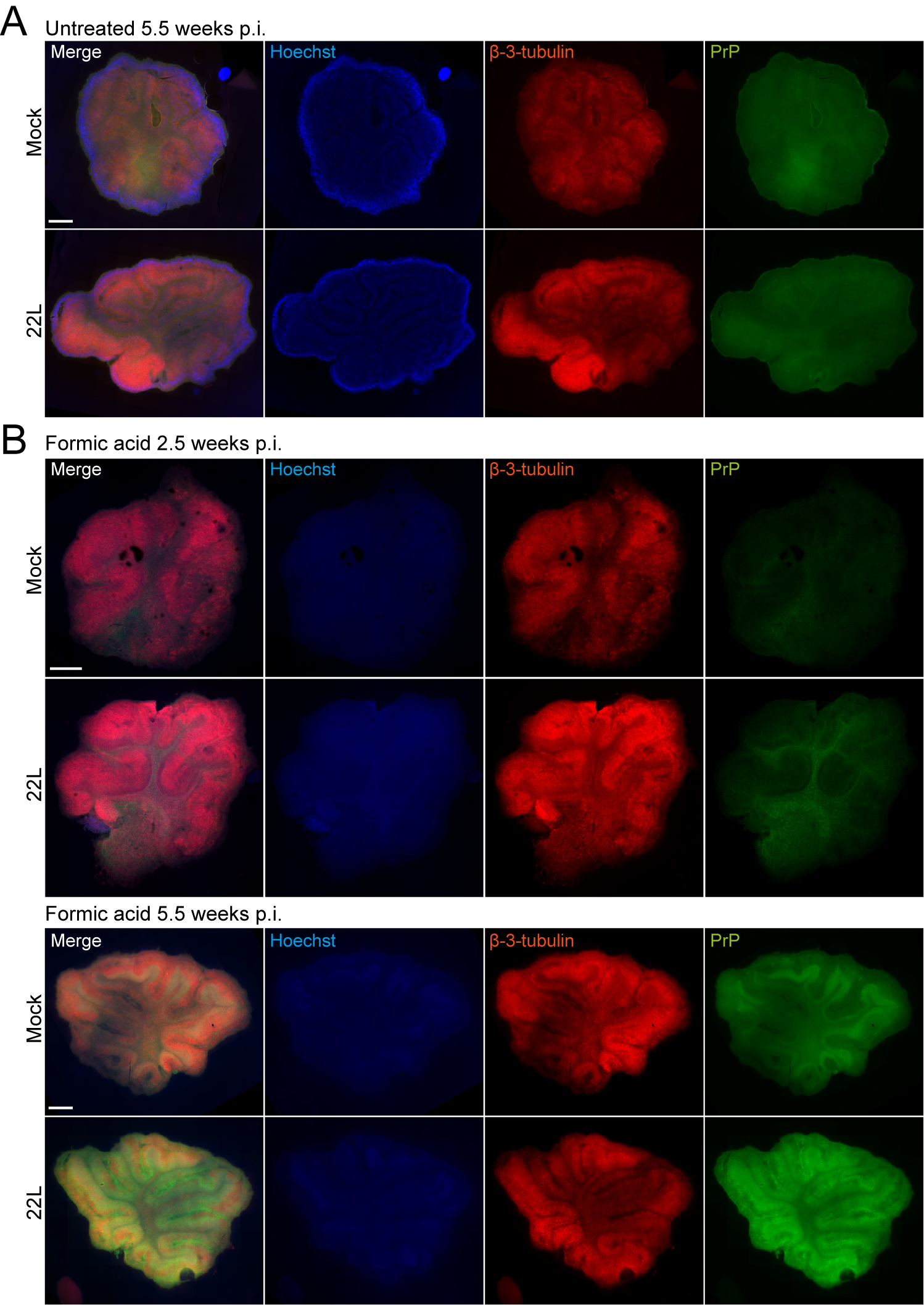


Supplementary Figure 1. Antigen denaturation by formic acid. Cerebellar slices exposed to Mock or infected with 22L brain homogenate were cultured for 2.5 or 5.5 weeks. Slices were fixed, permeabilized and (A) left untreated or (B) were treated with > 98% formic acid for the detection of PrP^d^ with mAb 4H11 (green). Neurons were stained with pAb anti-β-3-tubulin (red) and nuclei were counterstained with Hoechst. Samples were analyzed by epifluorescence microscopy using the tile scanning function with identical imaging settings for each sample group. Scale bar: 500 μm.

**
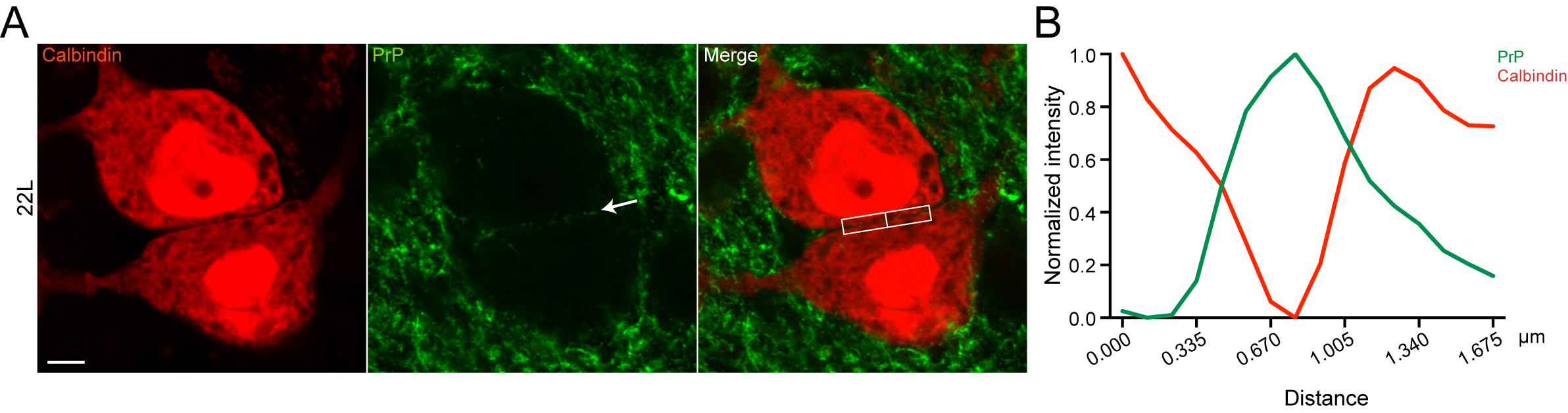
Supplementary Figure 2**. **Extracellular PrP^d^ deposition.** **(A)** Confocal microscopy analysis of PrP^d^ deposition in a 22L prion infected slice 9 weeks post infection. Purkinje cells were stained with anti-calbindin and PrP^d^ with anti-PrP antibody. The arrow marks PrP^d^ deposition in the region between adjacent PrP^d^ negative Purkinje cells. Scale bar: 5 μm. **(B)** Staining for PrP^d^ (green) and calbindin (red) displayed as a profile of normalized fluorescence intensities (arbitrary units). To demonstrate the presence of extracellular PrP^d^, intensity profiles of PrP^d^ and calbindin signals were measured along the width of the box (1.675 μm) and averaged along the length (11 μm) of the indicated region (box in A). The means of the fluorescent intensities were normalized using the formula I_norm_= (I-I_min_)/ (I_max_-I_min_). Quantitation was performed using Fiji software.
